# Factors influencing the crystallization of monosodium urate: a systematic literature review

**DOI:** 10.1186/s12891-015-0762-4

**Published:** 2015-10-14

**Authors:** Ashika Chhana, Gerald Lee, Nicola Dalbeth

**Affiliations:** Bone & Joint Research Group, Department of Medicine, University of Auckland, Auckland, New Zealand

**Keywords:** Urate solubility, Crystallization, Nucleation, Crystal growth, Gout

## Abstract

**Background:**

Gout is a chronic disease of monosodium urate (MSU) crystal deposition. Although hyperuricaemia is the central risk factor for development of gout, not all people with hyperuricaemia have subclinical MSU crystal deposition or indeed, symptomatic disease. The aim of this systematic literature review was to identify factors that contribute to MSU crystallization.

**Methods:**

A search was conducted of the electronic databases PubMed, Science Direct and Scopus. Articles were included if they contained original data related to MSU crystallization. The methods and results were summarized and categorized into articles describing at least one of the three key steps in MSU crystallization (reduced urate solubility, nucleation and growth).

**Results:**

A total of 2175 articles were initially identified in our systematic search with 35 of these articles included in the final analysis. Elevated urate concentration was identified as a central factor driving all three stages of MSU crystallization. Factors that were found to consistently reduce urate solubility were reduced temperatures, pH 7–9 and various ions including sodium ions. Connective tissue factors including bovine cartilage homogenates and healthy human synovial fluid and serum all enhanced urate solubility. MSU nucleation was found to be increased by a number of factors, including sodium ions, uric acid binding antibodies, and synovial fluid or serum from patients with gout. Other than elevated urate concentrations, no other specific factors were identified as promoters of MSU crystal growth.

**Conclusions:**

Increased urate concentration is the key factor required at each stage of MSU crystallization. Different proteins and factors within connective tissues may promote MSU crystallization and may be important for determining the sites at which MSU crystallization occurs in the presence of elevated urate concentrations.

**Electronic supplementary material:**

The online version of this article (doi:10.1186/s12891-015-0762-4) contains supplementary material, which is available to authorized users.

## Background

Gout is a chronic disease of monosodium urate (MSU) crystal deposition. The clinical features of gout occur due to host tissue responses to these crystals [1]. Four phases or stages of disease have been proposed [2, 3]: A: asymptomatic hyperuricaemia, without evidence of MSU crystal deposition; B: asymptomatic hyperuricaemia and evidence of MSU crystal deposition (by microscopy or advanced imaging); C: MSU crystal deposition with prior or current symptoms of acute gout flares; D: advanced gout (tophi, chronic gouty arthropathy, bone erosion).

Hyperuricaemia is the central risk factor for development of gout [4]. However, many people with hyperuricaemia do not have subclinical MSU crystal deposition or indeed, symptomatic disease. For example, a recent dual energy computed tomography study has shown that only 24 % of asymptomatic individuals with serum urate concentrations >9 mg/dL had imaging evidence of MSU crystal deposition [5]. Similar findings have been reported in ultrasonography studies of individuals with asymptomatic hyperuricaemia [6–8]. A further important observation is that MSU crystal deposition occurs preferentially at certain sites, particularly the 1st metatarsophalangeal joint, femoral condyle, Achilles tendon, and patellar tendon [9, 10]. Collectively, these data suggest that factors in addition to urate concentration contribute to MSU crystallization.

Viewed microscopically, MSU crystals are needle-shaped with a triclinic structure containing three unequal axes, none of which are perpendicular to the others [11, 12]. At the molecular level, the long axis of a three-dimensional MSU crystal is made up of sheets of closely spaced purine rings orientated parallel to the (011) plane. These sheets are stacked one on top of the other. Each purine ring contains urate anions aligned closely together through hydrogen bonding, and water molecules which are held in place by coordination to two sodium ions and by one hydrogen bond to the purine ring. The stacking interactions between the sheets and interlayer coordination to sodium ions results in twisting of the urate ion 7.7° out of the (011) plane. These interactions are required for urate ions to maintain octahedral geometry about the sodium ion [11, 12].

In general, three keys steps are required for crystal formation from a liquid mixture [13]; reduced solubility (leading to supersaturation), nucleation (which involves formation of clusters of solute molecules that ultimately reach a critical size and become stable) and crystal growth (subsequent growth of stable nuclei). Supersaturation drives both nucleation and growth of crystals, and controls the rate of crystal formation [13]. Using this general framework of crystal formation, we performed a systematic literature review with the aim of identifying factors that contribute to MSU crystallization in gout.

## Methods

**Fig. 1:**
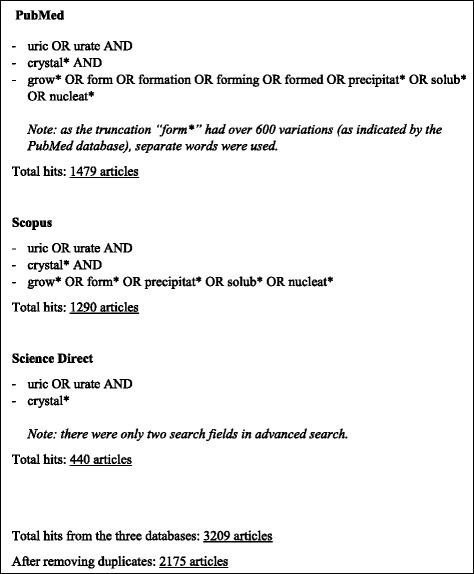
A summary of the search strategy used to identify articles related to the crystallization of MSU and results from database searches

**Fig. 2:**
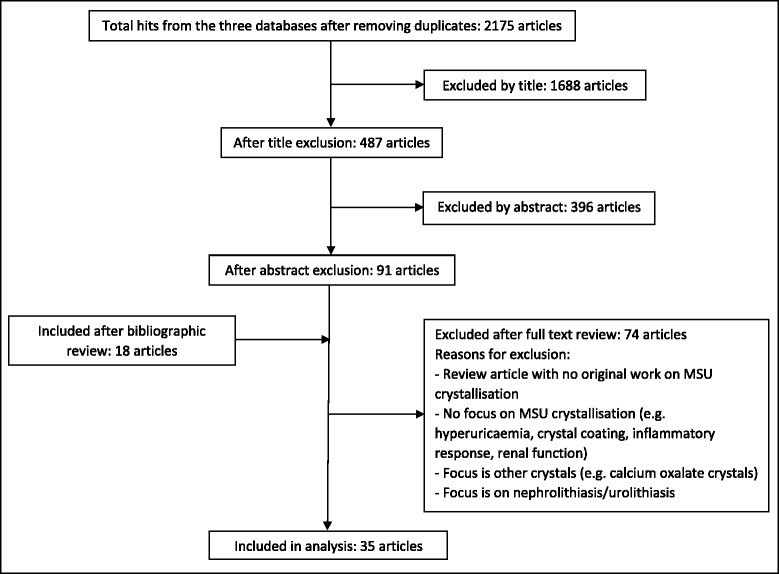
Flow diagram for selection of articles included in the review

A systematic search strategy was formulated to identify factors that contribute to MSU crystallization. This analysis was conducted in concordance with Preferred Reporting Items for Systematic Reviews and Meta-analyses (PRISMA) guidelines [14]. Electronic searches were performed in the following online databases: PubMed, Science Direct and Scopus. The following search keywords were used: “uric”, “urate”, “crystal*”, “grow*”, “form*”, “precipitat*”, “solub*” and “nucleat*”. The PubMed database indicated that the truncation “form*” had over 600 variations and omitted some search results when this truncation was used. For this reason, search words used instead of “form*” in the PubMed search were “form”, “formation”, “forming” and “formed”. An example of the search strategy is shown in Fig. 1. Data sources were English publications from these databases. No date restrictions were used (earliest database search date was 1946). The search was completed on 1st December 2014. Articles were included if they contained original data related to MSU crystallization. Congress abstracts were not searched or included in the current analysis. Exclusion criteria were: review article with no original work or data on MSU crystallization; not in English; no focus on MSU crystallization (e.g. focused on mechanisms of hyperuricaemia, crystal coating, the inflammatory response or renal function); primarily focused on other types of crystals; or related to uric acid nephrolithiasis or urolithiasis. Duplicate articles were removed from the search list. Articles were then excluded by review of titles and abstracts by two independent reviewers (AC and GL). For the remaining articles, the full text was reviewed to identify articles that met the inclusion criteria. Bibliographic references of individual publications were also identified and reviewed during this stage (Fig. 2).

Each article that fulfilled inclusion criteria was then assessed by two independent reviewers (AC and GL). The methods and results were summarized and categorized into articles describing one or more of the three key MSU crystallization steps (solubility, nucleation and growth). The specific factors assessed (e.g. pH, temperature, connective tissue factors and proteins, concentration of ions, antibodies, kinetics, crystal morphology and other), and the types of assay used in each study (in vitro, ex vivo, in vivo) were recorded.

A quality score was also determined. As a quality score has not been described for systemic literature reviews of laboratory studies, we devised the following score expressed as a number out of 4, determined by the sum of four questions (each positive answer scored as 1): was there a clear statement of the aims of the research?; is the method appropriate for the study aims?; are the results stated consistent with the results presented? i.e., do the figures, or data presented support the written results?; and do the results/data justify the conclusions made?

## Results

### Search results

Following removal of duplicates, 2175 journal articles were identified from the three databases searched. Titles were reviewed and 1688 articles were excluded because the titles were either not in English, or did not relate to the research question. Abstracts were then reviewed and further exclusion of articles was performed using the same criteria as above. From the bibliographic review, 18 additional articles were identified. In total, a full review of 109 journal articles was completed independently by the two reviewers. Of these articles, 35 were identified as original studies related to gout and MSU crystallization (Figs. 1 and 2). Of those studies included in the final analysis, 19 investigated urate solubility, 21 investigated MSU crystal nucleation, and 9 examined MSU crystal growth (Additional file 1: Table S1).

The type of assay or assays used to assess each stage of MSU crystal formation is shown in Table 1. Most studies used in vitro assays involving the crystallization of MSU from supersaturated solutions of urate (34 articles). Ex vivo assays generally involved the addition of synovial fluid or serum from healthy people or patients with various arthropathies, including gout (10 articles). Very few assays were performed in vivo and these were done in humans (1 article), rabbits (2 articles) or mice (1 article). Some studies used more than one type of assay.

**Table 1:** The different assays used in studies of MSU crystallization. These criteria were used to categorize each article into one or more of the main MSU crystallization steps

Crystallization category	Assay
Solubility	Two different methods were used to measure the solubility of MSU in vitro. The first method involved changing the conditions of the solvent, or adding specific factors to the solvent and then measuring the change in urate concentration. The second method measured the maintenance of MSU in crystalline form whereby one or more synthesized MSU crystals (seed crystals) were added to the solvent and the dissolution of these crystals was measured. The solvents used for both types of assays were usually solutions of sodium urate or water.
Nucleation	Nucleation assays were typically done in supersaturated sodium urate solutions. Various factors were added to the system and endpoints included whether new crystals formed in the presence of the given factor or change in condition, the time taken to form new crystals, and measurement of the total weight of crystals formed at the end of the experiment. Any studies that used MSU seed crystals in the assay for measuring crystal weight were assigned to this category.
Crystal growth	Crystal growth assays involved exposing one or more MSU seed crystals to a solution of sodium urate and measuring the rate of growth of the seed crystals over time and/or the change in length of the crystals. Changes in crystal morphology in the presence of a given factor were typically examined using microscopy.

In the quality assessment of included articles, the majority of articles were given the maximum quality score of 4. Seven articles were given a score of 3, and one article was given a score of 2. Those articles with a score of 2 or 3 generally did not fully explain methodology used, included results in the text that were not presented as data or images; and/or the results shown did not fully support or justify the written text or conclusions made.

Four of the articles included in the final analysis were published within the past two decades (1996–2015), 20 articles were published from the previous two decades (1976–1995), and 11 articles were published prior to 1976.

### Factors affecting urate solubility

Solubility is the property of a solid solute to dissolve in a liquid to form a homogenous solution of the solute in the given solvent [15]. The saturation concentration of a substance can be obtained experimentally by determining the maximum amount of the substance that is soluble at a given temperature [13]. A supersaturated solution can be obtained by dissolving more substance than could normally be dissolved by the solvent by altering the conditions of dissolution, for example by changing the pH or temperature of the solution [15].

For this analysis, in the context of MSU crystallization, those studies that investigated factors that altered urate concentration (i.e., changed the concentration of solute), or factors that affected the dissolution of pre-existing MSU crystals (i.e., changed the saturation point of the solute) were assigned to the “solubility” category (Table 1). Using these assay criteria, there were 19 articles that examined the solubility of MSU. The results from these articles demonstrated that temperature, pH, concentration of ions, proteins and various connective tissue factors all affected the solubility of MSU.

#### Temperature

Generally two methods were used to determine the solubility of urate at different temperatures. First, excess uric acid was dissolved in solutions of sodium chloride or water at different temperatures and uric acid concentration was measured once equilibrium had been reached [16–21]. Second, MSU crystals formed on microscope slides were slowly heated using a specialized temperature-controlled microscope stage and the temperature at which the crystals dissolved was used to calculate solubility [18, 22]. Although, this second method may have underestimated the solubility, as the heating rate was too fast for equilibrium to be established [21]. In any case, both methods demonstrated that decreasing temperatures led to reduced solubility of urate in sodium chloride or water solutions. Loeb [19] calculated the solubility of urate was 6.8 mg/dL at physiological temperature and sodium levels, based on in vitro data produced by Allen et al. [16].

#### pH

Similar to the temperature experiments, in vitro methods were used to determine the effect of pH on MSU solubility. In one method, supersaturated solutions of sodium urate at different pH levels were left to equilibrate and uric acid concentrations were used to calculate solubility. The results from these studies were variable, but urate solubility was reported to be greater at pH levels ≤6 or ≥10, with minimal solubility observed at pH 7–8 [18, 20]. The reason for increased urate solubility at either very high or very low pH levels may be due to the different species of urate that existed in these conditions. Theoretical equations were used to determine the proportion of uric acid-urate species in solution at 37 °C at different pH levels [18]. This study proposed that at low pH levels, fully protonated uric acid was the main species present, and at higher pH levels, urate ions were the predominant species present. The switch of uric acid to its mono-protonated urate form was further elucidated by x-ray diffraction in 1981 [23]. In this experimental study, supersaturated solutions of sodium urate at different pH levels were maintained at 37 °C and the fractions of uric acid, biurate and urate ions measured. At pH 5.62, a switch occurred whereby the predominant species present was urate, compared to pH <5.62 where uric acid was the predominant species present. At approximately pH 9, urate ions began to transform to biurate ions [23]. Mono-protonated urate ions were identified as the ion species incorporated into MSU crystals at physiological pH levels [12].

#### Concentration of ions

The most important factor for determining urate solubility was the presence of excess urate ions within the solution. In vitro experiments showed that the rate of dissolution of pre-existing MSU crystals was inversely proportional to the degree of urate saturation [23, 24]. However, the concentrations of other ions present within the solution also affected urate solubility. Sodium ions reduced urate solubility at physiologically relevant concentrations [16, 17, 20, 25]. Furedi-Molhofer et al. [26] showed that at pH 7.5 and 35 °C, the addition of increasing concentrations of sodium chloride to undersaturated solutions of sodium urate caused the phase boundaries of urate solubility to shift towards lower urate concentrations [26]. Other cations, such as K^+^, Mg^2+^, NH4^+^, Ca^2+^ and Cu^2+^ ions, also reduced urate solubility to varying degrees, although these findings may have been influenced by changes in pH that were not always taken into account during analysis [25, 27].

#### Connective tissue factors and proteins

There was substantial evidence to suggest that factors derived from cartilage, synovial fluid and serum altered urate solubility in both in vitro and ex vivo assays. Acetone dried homogenates of bovine nasal cartilage markedly enhanced urate solubility at 4 °C, compared to homogenates derived from heart, liver, brain or kidney tissue [28]. Further analysis suggested that protein polysaccharides within the cartilage were responsible for this effect and that the structural integrity of these proteins was vital for their effect on urate solubility [28]. Other cartilage matrix components also altered urate solubility in vitro. Addition of proteoglycans extracted from porcine cartilage to supersaturated solutions of sodium urate resulted in increased urate solubility. Similarly to the polysaccharides, only structurally intact, aggregated proteoglycans enhanced urate solubility; non-aggregated proteoglycans treated with enzyme did not change urate solubility [29].

There was conflicting evidence for the direction of the effect of isolated glycosaminoglycans on urate solubility. In one study at 37 °C, increasing concentrations of chondroitin-4-sulphate (63–188 mg/mL) reduced urate solubility [30]. However, another study that used a similar concentration of chondroitin-4-sulphate (80 mg/mL) showed that there was a slight increase in urate solubility at 4 °C [28]. A third study reported that very low concentrations of chondroitin-4-sulphate (0.1–0.4 mg/mL) also slightly increased urate solubility at 37 °C [31]. The different experimental conditions and variation in the source of chondroitin sulphate used in these experiments made these results difficult to interpret.

Factors derived from other connective tissues such as plasma and synovial fluid also influenced urate solubility. Multiple studies examined the effect of synovial fluid from people with or without arthritis, including gout and rheumatoid arthritis (RA) [20, 27, 32]. In healthy people, urate solubility was lower in plasma and synovial fluid compared to urine [20]. Dialysis of the plasma prior to completing solubility assays further enhanced the reduction effect on urate solubility, which suggested that there was a macromolecule present within plasma that enhanced urate solubility, possibly by sequestering urate [20]. Albumin was one potential macromolecule reported to influence urate solubility within plasma. Human serum albumin significantly enhanced urate solubility at 26 °C and 37 °C [20]. However, other studies reported only very minor increases in urate solubility with human or bovine serum albumin at similar or even higher concentrations [28]. Articles also reported conflicting data regarding the influence of hyaluronate in determining urate solubility. One in vitro study reported that low concentrations of hyaluronic acid slightly enhanced urate solubility [31]. While another study reported that treatment of synovial fluid with hyaluronase resulted in enhanced MSU solubility [32], in which case it would be expected that intact hyluronate reduces urate solubility. In samples from patients with osteoarthritis, RA or gout, the dissolution of MSU crystals was generally greater in plasma compared to synovial fluid in all groups, with the smallest difference between plasma and synovial fluid observed within the gout group [32]. Another study showed that synovial fluid from one patient with gout had reduced urate solubility to a greater extent than synovial fluid from one patient with RA [27].

### Factors affecting MSU nucleation

Nucleation is the appearance of new crystals in a system. Supersaturation alone is not sufficient to cause crystallization and before crystallization can actually occur, a number of minute solid bodies or “nuclei” must exist within the solution to act as centres of crystallization [33]. In MSU crystallization, it was hypothesized that nucleation in vivo occurs after MSU molecules have clustered together and reached a critical mass or size, whereby they are stabilized and no longer susceptible to dispersion forces within the solvent which would normally promote dissolution [34, 35]. In our analysis, those studies that reported to measure nucleation or precipitation were categorized into this stage of crystallization. Nucleation assays were typically done in supersaturated sodium urate solutions and endpoints included whether new crystals formed in the presence of a given factor or change in condition, the time taken to form new crystals, and measurement of the total weight of crystals formed at the end of the experiment (Table 1). Any studies that used MSU seed crystals in the assay for measuring crystal weight were categorized into “nucleation” rather than “growth”, as these experiments cannot differentiate between newly formed crystals and growth of existing seed crystals. Only those studies that measured crystal length were categorized into the growth section.

#### Concentration of ions

Highly elevated urate concentrations in vitro were crucial for MSU crystal nucleation, with a greater number of MSU crystals formed as the concentration of urate increased [12, 36, 37]. In addition, there were other ions that influenced MSU nucleation. In solutions of supersaturated sodium urate (10–12 mM), the addition of increasing sodium ions to the solution resulted in a greater number of MSU crystals formed in a dose-dependent manner [37]. Similarly, as urate concentration was reduced, a greater concentration of sodium ions was required for spontaneous nucleation to occur [36]. At physiological concentrations, K^+^, Mg^2+^ and Cu^2+^ ions slightly reduced MSU nucleation (number of crystals formed after 1 month) [22, 27, 36]. The effect of calcium ions on MSU nucleation was not clear. It was initially reported by Khalaf et al. [22] and Wilcox et al. [27] that Ca^2+^ ions significantly enhanced MSU nucleation [22, 27]. However, the same authors later reported that there was no change in the number of MSU crystals formed in a supersaturated solution of sodium urate in the presence of additional Ca^2+^ ions, and further analysis revealed that the crystals formed in this case were actually calcium urate crystals and not MSU crystals [36].

#### Connective tissue factors and proteins

Given that MSU crystals preferentially form and deposit on cartilage and are found in biological fluids where there are multiple different proteins and molecules present, it seems likely that MSU nucleation in vivo occurs in a primary heterogeneous manner (induced by foreign particles). A number of studies reported that synovial fluid taken from people with gout enhanced MSU crystal nucleation in ex vivo nucleation assays. In particular, addition of gouty synovial fluid to supersaturated solutions of sodium urate resulted in a faster time to appearance of MSU crystals and a greater total weight of MSU crystals formed, compared to synovial fluid from healthy people and patients with RA or other crystal arthropathies [22, 27, 37, 38]. These results were independent of the baseline uric acid levels in the synovial fluid samples [37]. These findings suggested that there was a factor present in gouty synovial fluid that acted as a promoter of MSU nucleation in hyperuricaemic conditions.

Addition of low concentrations of serum (0.25–6 %) also increased the amount of MSU crystals formed from supersaturated solutions in vitro [38, 39], although no differences between gouty versus healthy human serum have been reported [40]. A more detailed in vitro analysis suggested that there was a high molecular weight, heat sensitive protein present in serum that enhanced MSU crystallization [38]. Human and bovine serum albumin, globulins (particularly γ-globulin) and collagen type I, all increased MSU nucleation to varying degrees [38, 41, 42]. Carboxylate groups present on human albumin were required for the positive effect of albumin on MSU nucleation [41].

A number of observations have implicated damaged cartilage or tissue factors derived from cartilage as promoters of MSU nucleation [43, 44]. In this review, most studies that investigated the effects of cartilage factors on MSU crystallization examined changes in urate solubility as an endpoint. Only one study of cartilage factors met our criteria for a nucleation assay. Perl-Treves et al. [41] reported that chondroitin sulphate and hyaluronic acid both had no effect on the time to nucleation in supersaturated solutions of sodium urate at pH 8.0 [41].

#### Antibodies

More recent articles reported the presence of specific uric acid binding antibodies that promoted MSU crystal nucleation, possibly through stabilization of MSU nuclei [45–47]. In one study, IgG antibodies were isolated from synovial fluid from people with gout and other arthropathies such as RA and osteoarthritis. In vitro, the antibodies isolated from gouty synovial fluid had a much faster rate of MSU crystal appearance in nucleation assays compared to other synovial fluid samples [46]. Another study demonstrated that IgG antibodies isolated from the serum of rabbits injected with MSU crystals significantly increased nucleation (faster time to appearance of crystals) in supersaturated sodium urate solutions in vitro. Antibodies isolated from rabbits injected with other types of crystals did not have the same effect on MSU nucleation and the authors suggested that this indicated high specificity between the antibody binding sites and the crystal surface, and the uric acid binding antibodies were acting as nucleating templates for MSU crystallization [47]. Kanevets et al. [45] showed that serum collected from mice injected with MSU crystals contained antibodies that bound to MSU crystals ex vivo; 85 % of the bound antibodies were IgM and 14 % were IgG antibodies. Further analysis revealed that the F(ab’)_2_ domain and pentameric structure of IgM antibodies were important for this binding and subsequent MSU nucleation [45]. While nucleation was not specifically examined in vivo in this study, mice were injected with uric acid with either uric acid binding antibodies or control antibodies, and blood uric acid levels were measured over time as an indirect method for measurement of MSU crystal formation. From this experiment, the concentration of uric acid in the blood was significantly reduced post-injection with the uric acid binding antibodies compared to control antibodies, which suggested that MSU nucleation had occurred [45].

#### Other factors

As well as primary nucleation, it is likely that secondary nucleation also occurs during MSU crystallization. That is the nucleation of new crystals from already formed seed crystals [33]. Images of branch points on MSU crystals obtained by scanning electron microscopy supported the notion of secondary nucleation [12]. In vitro, the addition of MSU seed crystals to a supersaturated solution of sodium urate increased the rate of MSU formation over time in a dose-dependent manner [39, 48]. Lead urate seed crystals also induced MSU nucleation in vitro [49, 50]. Seed crystals of other types (silica, CPPD and hydroxyapatite) had no effect on MSU nucleation [39], suggesting that MSU nucleation is not induced by all types of particulates, there is some specificity.

Various chemical dyes have also been tested in vitro for their effects on MSU nucleation. Neutral red dye and methylene blue both increased time to crystallization in nucleation assays [21, 23, 51].

### Factors affecting MSU crystal growth

Assays that measured the change in length of MSU crystals over time or those that examined the morphology of MSU crystals grown in the presence of additional factors were categorized into the “growth” section (Table 1).

#### Concentration of ions

The rate of MSU crystal growth over time was dependent on the level of urate saturation [21, 51]. Allen et al. [16, 17] measured the growth of a single fixed MSU crystal over time in supersaturated solutions of urate at different concentrations. In this experiment, crystal length and time had a linear relationship when temperature and urate concentration were kept constant. As the concentration of urate increased, the rate of crystal growth also increased [16, 17]. At the molecular level, Perrin et al. [39] adsorbed MSU crystals to coverslips and solutions of supersaturated urate were flowed over the crystals at physiological pH, temperature and sodium concentration [12]. Atomic force microscopy was used to visualize growth on the (010) crystal surface. Solutions with less than 4 mM urate led to dissolution of MSU crystals, whereas solutions with 4 mM urate resulted in stability, with no further growth of the crystals and no dissolution. Between 4 and 8 mM urate, consistent growth of crystals was observed. At 7 mM urate, macroscopic islands approximately 2 μm length × 175 nm height were observed on the (010) surface. These islands grew in a direction dependent manner and the time for incorporation of the island onto the crystal was dependent on the island size and the level of supersaturation. This experiment also demonstrated that these islands were able to re-orientate themselves on the crystal surface if a growth hillock (physical obstruction) was encountered [12].

#### Connective tissue factors and proteins

Compared to urate solubility and MSU crystal nucleation, the effects of connective tissue factors and proteins on MSU crystal growth have not been studied extensively. One study compared the length of MSU crystals from ex vivo tophus samples to MSU crystals grown in vitro in the presence of synovial fluid or serum components [52]. In vitro, MSU crystals grown in the absence of any additional factors had a wide range of lengths (93 ± 43 μm); whereas crystals grown in the presence of human serum or synovial fluid had a much tighter range of crystal lengths (serum, 35 ± 12 μm; synovial fluid, 36 ± 10 μm) which was more comparable to sizes found within tophi (11 ± 3 μm), although still longer. The addition of γ-globulin produced a tighter range of crystals lengths, while chondroitin sulphate, α- and β-globulins, and human serum albumin all resulted in a wider range of crystal lengths [52]. Human serum albumin also increased the thickness of MSU crystals grown in vitro [41].

## Discussion

**Fig. 3:**
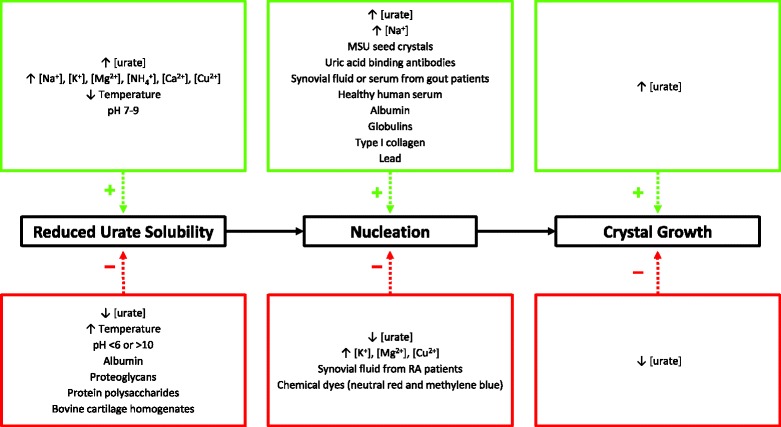
Summary of the factors that influenced the three stages of MSU crystal formation, as identified in the systematic literature review

This analysis has identified elevated urate concentrations as the main factor required for the three stages of MSU crystallization, including reduced urate solubility, MSU nucleation and MSU crystal growth. Urate solubility was also shown to be significantly influenced by various ions including sodium ions, temperature and pH, with colder temperatures and slightly basic conditions shown to be the ideal environment for MSU crystallization to occur. Uric acid binding antibodies, globulins, collagen, lead and human serum or synovial fluid were all shown to promote MSU crystallization at the nucleation stage. Increased urate concentration was the only factor identified in this analysis as a specific promoter of MSU crystal growth (Fig. 3).

It is currently not known why MSU crystals form in some hyperuricaemic individuals but not others. In patients with MSU crystal deposition and gout, there must be other factors present within the serum or joints of these individuals that promote MSU crystallization in the presence of elevated tissue urate concentration. Indeed, some of the studies included in this analysis indicate that the synovial fluid from these patients does reduce urate solubility and enhance MSU nucleation [20, 22, 27, 32, 37, 40]. However, the number of samples assessed was usually small, sometimes with only one patient sample examined. In addition, for the urate solubility studies, synovial fluid and serum samples from healthy individuals were not assessed and compared to samples taken from patients with gout [27, 32]. Therefore, it is still not clear whether all human synovial fluid and serum reduces urate solubility, or if this effect is specific to synovial fluid and serum taken from patients with gout or other forms of arthritis. Studies using samples from a greater number of people, including healthy individuals, will provide more insight.

High urate concentration was found to be crucial for driving MSU crystallization at all three stages (reduced solubility, nucleation and growth). No other factor was found to influence all three stages of crystallization, although this is not necessarily because no other factor has a role in each stage, but simply because all other factors have not been studied as extensively. Most studies investigated changes in urate solubility or MSU nucleation.

While most of the evidence published was very robust and there was general agreement between studies, there were some factors tested that have had conflicting results reported, particularly for connective tissue factors and proteins, such as glycosaminoglycans, albumin and hyaluronate [20, 28, 30–32]. Different protein sources, experimental conditions and methods may account for these discrepancies. In addition, some concentrations used in the in vitro assays were quite low or may not have reflected physiological concentrations; use of higher concentrations may have led to different results [41].

Most of the studies described in this analysis used in vitro methods to study MSU crystallization and typically only investigated one change in condition or factor at a time. However, in vivo, urate molecules and MSU crystals are surrounded by connective tissue factors and proteins, as well as other biological factors, such as complement proteins (important for the inflammatory response to MSU crystals) and antibodies. The interactions between these factors may also have a role in MSU crystal formation.

The preference for MSU crystals to form on cartilage surfaces is well documented [44] and joints affected by previous trauma or osteoarthritis are more likely to be affected by MSU crystal deposition [43]. Consistent with these clinical observations, many factors and proteins within connective tissues were found to influence urate solubility and MSU nucleation. The physical structure of connective tissues may also influence MSU crystallization and growth. Histological studies have shown that MSU crystals are present within the joint aligned to collagen fibres within the tendon in a highly organized manner [53]. Similarly, crystals identified within cadaveric cartilage from a patient with gout using scanning electron microscopy were arranged in specific bow-shaped bundles [54]. In the current review, the addition of various connective tissue factors and proteins to in vitro growth assays was shown to influence MSU crystal lengths [41, 52], although the direction of these effects were difficult to interpret as the main focus of these studies was to determine the variation in crystal lengths with these additional factors, rather than whether or not these factors were specifically promoting or inhibiting MSU crystal growth. Collectively, these findings suggest that the physical environment in which MSU crystallization occurs may influence growth patterns.

In vitro studies have shown that urate solubility is lowest in slightly basic conditions [18, 20]. This seems somewhat contradictory to the clinical observation that cartilage surfaces are slightly acidic in osteoarthritic joints, and these surfaces become more acidic as cartilage destruction progresses [55]. At the reported pH of human osteoarthritic cartilage (pH 5.5–6.2) [55], urate solubility is actually still very low and mono-protonated urate ions remain the predominant species present within this pH range [23], suggesting that MSU crystallization does still occur in these slightly acidic conditions. In addition, enzymes such as cathepsin K are specifically activated at lower pH values and lead to further degradation of cartilage in osteoarthritic joints [55–57]. This increased enzyme activity is likely to result in a local increase in matrix breakdown products which may serve as MSU crystal nucleation sites, further promoting MSU crystallization at sites of osteoarthritis. A systematic analysis of the effect of cartilage tissue and specific cartilage factors on MSU nucleation would provide more insight as to why MSU crystals preferentially form and deposit on cartilage surfaces, particularly those affected by osteoarthritis.

One of the most interesting findings of this review has been that very few studies related to MSU crystallization have been published in the last 20 years. This result suggests that research into the crystallization of MSU has not been an active area of gout research in recent times. The continuation of this work using more recent advanced imaging technologies and laboratory methods will be of great benefit to further improve our knowledge of why and how MSU crystallization occurs in patients with gout.

## Conclusion

In this systematic literature review of factors involved in the crystallization of MSU, elevated urate concentration was consistently identified as the key factor required for all three stages of MSU crystallization. Other factors shown to be important for controlling urate solubility included sodium ions, colder temperatures and slightly higher pH levels. Increased MSU nucleation has been reported in the presence of human serum or synovial fluid and various isolated connective tissue proteins. Other than locally increased urate concentrations, no additional factors were identified as specific promoters of MSU crystal growth. Further research examining the role of connective tissues in MSU crystallization will ultimately enhance our understanding of the biological basis of gout and may give insight into why only a subset of people with hyperuricaemia eventually develop gout.

## Additional file

Additional file 1: Table S1. Summary of journal articles included in the review categorized by the relevant stage of crystallization. (DOCX 37 kb)
