# Supplementary material for: Factors influencing the crystallization of monosodium urate: a systematic literature review
Source: BMC Musculoskelet Disord. 2015 Oct 14;16:296. doi: 10.1186/s12891-015-0762-4 (PMC4606994; doi:10.1186/s12891-015-0762-4)
Supplement: Additional file 1: Table S1. — Summary of journal articles included in the review categorized by the relevant stage of crystallization. (DOCX 37 kb) [file 12891_2015_762_MOESM1_ESM.docx]

**ADDITIONAL FILE 1**

**Table S1:** **Summary of journal articles included in the review categorized by the relevant stage of crystallization**.

| Title | Author(s) | Year | Stage of crystallization | Type of assay | Factors examined | Quality Score/4 |
| --- | --- | --- | --- | --- | --- | --- |
| Solubility of sodium urate in the presence of chondroitin-4-sulphate. | Laurent T.C. | 1964 | Solubility | In vitro | Connective tissue factors | 4 |
| The acute attack of gouty arthritis. | Seegmiller, J. E. | 1965 | Solubility | Ex vivo (humans)  In vitro | Concentration of ions  Connective tissue factors | 3 |
| The interaction of monosodium urate with connective tissue components. | Katz W.A., Schubert M. | 1970 | Solubility | In vitro | Connective tissue factors  Proteins | 4 |
| Solubility of uric acid and monosodium urate | Wilcox W.R., Khalaf A. | 1972 | Solubility | In vitro | pH  Concentration of ions  Temperature | 3 |
| The influence of temperature on the solubility of monosodium urate | Loeb J.N. | 1972 | Solubility | In vitro | Temperature | 4 |
| Local concentration of urate in the pathogenesis of gout | Simkin P.A. | 1973 | Solubility | In vivo (humans) | Diffusion rates of urate | 3 |
| Factors affecting urate solubility in vitro | Kippen I., Klinenburg J.R., Weinberger A., Wilcox W.R. | 1974 | Solubility | Ex vivo (humans)  In vitro | pH  Concentration of ions  Temperature  Connective tissue factors  Proteins | 4 |
| Enhancement of urate solubility by connective tissue. I. Effect of proteoglycan aggregates and buffer cation | Perricone E., Brandt K.D. | 1978 | Solubility | In vitro | Concentration of ions  Connective tissue factors | 4 |
| Physiological chemistry of uric acid: Solubility, colloid and ion-binding properties | McNabb R.A.,  McNabb F.M.A. | 1980 | Solubility | In vitro | pH  Concentration of ions  Proteins | 4 |
| Plasma and synovial fluid as solvents for monosodium urate | Dorner R.W., Weiss T.D., Baldassare A.R., Moore T.L., Zuckner J. | 1981 | Solubility | Ex vivo (humans)  In vitro | Connective tissue factors  Proteins | 4 |
| Solubility and nucleation of monosodium urate in relation to gouty arthritis | Khalaf A.A, Wilcox W.R | 1973 | Solubility  Nucleation | Ex vivo (humans)  In vitro | Concentration of ions  Temperature  Connective tissue factors  Additives (ethanol) | 3 |
| Nucleation of monosodium urate crystals | Wilcox W.R.,  Khalaf A.A. | 1975 | Solubility  Nucleation | In vitro | pH  Concentration of ions  Connective tissue factors  Additives (ethyl alcohol)  Mechanical stimulation  X-rays | 3 |
| The crystallization and dissolution of sodium urate | Lam Erwin C-Y., Nancollas G.H. | 1981 | Solubility  Nucleation | In vitro | pH  Kinetics  Additives (dyes)  Mechanical stimulation | 4 |
| Growth of monosodium urate monohydrate crystals: Effect of cartilage and synovial fluid components on in vitro growth rates | Burt H.M., Dutt Y.C. | 1986 | Solubility  Nucleation | In vitro | Connective tissue factors  Proteins | 4 |
| A role of IgM antibodies in monosodium urate crystal formation and associated adjuvanticity | Kanevets U., Sharma K., Dresser K., Shi Y. | 2009 | Solubility  Nucleation | In vivo (mice)  In vitro | Antibodies | 4 |
| Studies of urate crystallization in relation to gout | Fiddis R.W., Vlachos N., Calvert P.D. | 1983 | Solubility  Nucleation  Crystal growth | In vitro | Connective tissue factors  Proteins  Kinetics  Additives (dyes) | 3 |
| Precipitation of sodium acid urate from electrolyte solutions | Furedi-Milhofer H., Babic-Ivanicic V., Milat O., Brown W.E.,  Gregory T.M. | 1987 | Solubility  Nucleation  Crystal Growth | In vitro | Concentration of ions  Kinetics  Morphology | 4 |
| Crystal growth of sodium acid urate | Allen D.J., Milosovich G., Mattocks A.M. | 1965 | Solubility  Crystal growth | In vitro | Concentration of ions  Temperature  Kinetics  Morphology  Additives (ammonium compounds) | 4 |
| Inhibition of monosodium urate needle crystal growth. | Allen D.J., Milosovich G., Mattocks A.M. | 1965 | Solubility  Crystal growth | In vitro | Concentration of ions  Temperature  Kinetics  Additives (ammonium compounds, dyes) | 4 |
| Crystallization of monosodium urate and calcium urate at 37°C | Tak H.K., Wilcox W.R., Cooper S.M. | 1980 | Nucleation | In vitro | Concentration of ions | 4 |
| Studies on the nucleation of monosodium urate at 37°C | Tak H.K., Cooper S.M., Wilcox W.R. | 1980 | Nucleation | Ex vivo (humans)  In vitro | Concentration of ions  Connective tissue factors  Proteins | 4 |
| The effect of lead upon urate nucleation. | Tak H.K., Wilcox W.R., Cooper S.M. | 1981 | Nucleation | In vitro | Trace metal (lead) | 4 |
| Crystallization of monosodium urate monohydrate | Burt H.M., Dutt Y.C. | 1989 | Nucleation | In vitro | Kinetics | 4 |
| Evidence for a promoter of urate crystal formation in gouty synovial fluid | McGill N.W.,  Dieppe P.A. | 1991 | Nucleation | Ex vivo (humans)  In vitro | Connective tissue factors | 4 |
| The effect of biological crystals and human serum on the rate of formation of crystals of monosodium urate monohydrate in vitro | McGill N.W.,  Dieppe P.A. | 1991 | Nucleation | Ex vivo (human)  In vitro | Connective tissue factors  Kinetics  Seed crystals | 4 |
| The role of serum and synovial fluid components in the promotion of urate crystal formation | McGill N.W., Dieppe P.A. | 1991 | Nucleation | Ex vivo (humans)  In vitro | Connective tissue factors  Proteins | 4 |
| Antibodies against crystals | Kam M., Perl-Treves D., Caspi D., Addadi L. | 1992 | Nucleation | Ex vivo (humans and rabbits)  In vivo (rabbits)  In vitro | Antibodies | 4 |
| Specificity in the recognition of crystals by antibodies | Kam M., Perl-Treves D., Sfez R., Addadi L. | 1994 | Nucleation | In vivo (rabbits)  In vitro | Antibodies | 4 |
| Determination of urate crystal formation using flow cytometry and microarea X-ray diffractometry | Kaneko K., Maru M. | 2000 | Nucleation | In vitro | Proteins  Kinetics  Lactic acid | 4 |
| Structure of a lead urate complex and its effect on the nucleation of monosodium urate monohydrate | Sattar S., Carroll M.J., Sargeant A.A., Swift J.A. | 2008 | Nucleation | In vitro | Trace metal (lead) | 2 |
| Crystal growth of monosodium urate monohydrate | Calvert P.D., Fiddis R.W., Vlachos N. | 1985 | Nucleation Crystal growth | In vitro | Concentration of ions  Temperature  Proteins  Additives (dyes) | 3 |
| A structural approach to pathological crystallizations. Gout: The possible role of albumin in sodium urate crystallization | Perl-Treves D., Addadi L. | 1989 | Nucleation  Crystal growth | In vitro | pH  Proteins  Kinetics  Morphology  Polyelectrolytes | 4 |
| Monosodium urate monohydrate crystallization | Perrin C.M., Dobish M.A., Van Keuren E., Swift J.A. | 2011 | Nucleation  Crystal growth | In vitro | Concentration of ions  Kinetics  Morphology | 4 |
| Monosodium urate monohydrate, the gout culprit | Mandel N.S.,  Mandel G.S. | 1976 | Crystal growth | In vitro | Kinetics  Morphology | 4 |
| Morphological evidence for biological control of urate crystal formation in vivo and in vitro | Mcgill N.W., Hayes A., Dieppe P.A. | 1992 | Crystal growth | Ex vivo (humans) In vitro | Connective tissue factors Morphology | 4 |
